# Cross-Flow Filtration of *Escherichia coli* at a Nanofluidic Gap for Fast Immobilization and Antibiotic Susceptibility Testing

**DOI:** 10.3390/mi10100691

**Published:** 2019-10-12

**Authors:** Jan F. Busche, Svenja Möller, Matthias Stehr, Andreas Dietzel

**Affiliations:** 1 Institute of Microtechnology, Technische Universität Braunschweig, 38124 Braunschweig, Germany; svenja.moeller@tu-bs.de (S.M.); a.dietzel@tu-bs.de (A.D.); 2 Lionex GmbH, 38124 Braunschweig, Germany; mst@lionex.de

**Keywords:** antimicrobial resistance, microfluidics, cell capture, lab-on-a-chip, microcultivation, miniaturization

## Abstract

Infections with antimicrobial-resistant (AMR) bacteria are globally on the rise. In the future, multi-resistant infections will become one of the major problems in global health care. In order to enable reserve antibiotics to retain their effect as long as possible, broad-spectrum antibiotics must be used sparingly. This can be achieved by a rapid microfluidic phenotypic antibiotic susceptibility test, which provides the information needed for a targeted antibiotic therapy in less time than conventional tests. Such microfluidic tests must cope with a low bacteria concentration. On-chip filtering of the samples to accumulate bacteria can shorten the test time. By means of fluorescence microscopy, we examined a novel nanogap filtration principle to hold back *Escherichia coli* and to perform cultivation experiments with and without antibiotics present. Microfluidic chips based on the nanogap flow principle showed to be useful for the concentration and cultivation of *E. coli.* With a concentration of 10^6^ cells/mL, a specific growth rate of 0.013 min^−1^ and a doubling time of 53 min were achieved. In the presence of an antibiotic, no growth was observed. The results prove that this principle can, in future, be used in fast and marker-free antimicrobial susceptibility testing (AST).

## 1. Introduction

Antimicrobial-resistant (AMR) bacteria are currently one of the major threats in global health care. Due to the rampant use of antibiotics in animal fattening and human medicine, antibiotic resistance continues to spread across the globe [1]. The deaths caused by antibiotic-resistant infections were estimated to be as high as 700,000 in 2014. If no actions are taken, this number is expected to rise to 10 million people by 2050, overtaking the number of cancer deaths worldwide [2]. One key aspect to preventing these deaths is to ensure that reserve antibiotics retain their efficacy as long as possible. Rapid antibiotic resistance diagnostic procedures can make an important contribution to the correct and economical use of antibiotics. In addition, rapid resistance diagnostic procedures can accelerate the therapy of patients and reduce the risk of dying by sepsis, which is the main cause of death in intensive care units in hospitals [1]. If these devices are widely available and used, the unnecessary overprescription of broadband antibiotics could be avoided [3].

Currently, the available methods for antimicrobial susceptibility testing (AST) can be divided into genetic and phenotypic methods. Genetic ASTs are based on the analysis of the genome to find genes associated with antibiotic resistance found in a patient’s blood culture. While this can be achieved in around 1 to 2 h, it has the disadvantage that only known genetic markers can be detected, which are not always linked to phenotypic resistance behavior [4]. In addition, if no unknown mechanisms of resistance can be detected, this can lead to false negative results and thus the wrong medication given to the patient. When working with uncultured blood, the slightest contamination of the sample can lead to false positives when unrelated DNA is detected [5]. Due to these disadvantages of genetic assays, phenotypic assays are better suited for routine examinations. Phenotypic ASTs are based on the analysis of the growth behavior of bacteria in the presence of certain antibiotics in the culture medium. Conventionally, this is done in shaking cultures by measuring optical turbidity or on agar plates where the areas that are not overgrown by bacteria around antibiotic releasing surfaces are measured. This kind of resistance determination has the advantage that a resistance can be determined independently of the existing resistance mechanisms. These systems have the drawback that they need an overnight cultivation (24–72 h) before results can be evaluated [6]. To shorten this test duration, many new microfluidic-based phenotypic ASTs have been developed [7]. One approach is to combine a liquid bacterial culture with liquid agarose, which solidifies during cooling and holds the bacteria in a nutrient matrix. Antibiotics are then directed to the bacteria by diffusion and their minimum inhibitory concentration (MIC) is determined with single bacteria time-lapse imaging after 3 to 4 h [8]. This approach to diagnose antibiotic susceptibility is also used in other systems which obtained similar detection times (2.5 to 4 h) [9,10]. The detection principle however has the disadvantage of not providing concentration of bacteria. This leads to the fact that only bacterial samples with initial concentrations over 10^8^ cells/mL can be examined. Another attempt to build a fast AST is based on the mother machine design [11]. It has been adapted to allow better filling with bacterial samples and consists of 2000 cell traps. The testing principle relies on optical time-lapse observation with image analysis to detect bacterial growth inside these cell traps [12]. A disadvantage of this system is clogging, created by using a dead-end filter principle, which leads to challenges when dealing with polymicrobial infections [13]. By using a single droplet system, the MIC of *Escherichia coli* was measured by the metabolism of resazurin. However, only a bacterial concentration of 10^5^ CFU/mL could be measured [14]. In addition, gradient-based measurements were conducted [15]. Other groups use automated microdroplet systems to test combinations of antibiotics on bacteria [16,17]. While this method is very well suited for drug research [18], it is not so well suited for rapid AST, as in reality these high concentrations of bacteria are not always present in patient samples, which makes sample concentration necessary [14]. This restriction applies to several other systems where an external upstream filtration is needed [1]. Another method to investigate *E. coli* proliferation is by capturing them on membrane filters and real-time observation of expression dynamics via fluorescence microscopic detection of markers and therefore is not marker-free [19]. In another approach, the filtration is realized in 2.5 nL wells with stacked beads to lower the required minimum initial concentration in the sample to 10^5^ cells/mL for measuring the phenotypic response to antibiotics in 100 min [20]. The same applies to another system which only detects the fluorescent intensity [21]. In a further approach, bacteria are hydrodynamically captured in traps and the growth rate is examined individually in the presence of antibiotics. This system was tested with a concentration of 10^7^ CFU/mL [22]. By measuring electric fluctuations in a microfluidic channel, the antibiotic resistance of *E. coli* in the concentration range of 10^5^–10^7^ cells/mL could be measured [23]. Furthermore, centrifugal microfluidics enables the MIC determination of 10^6^ cells/mL *E. coli* suspensions [24]. In addition, the difference in MIC by the treatment of bacterial biofilm in comparison to fluid cultures has been investigated in microfluidics, concluding that higher concentrations of antibiotics are needed to treat bacteria grown in biofilms and not under planktonic conditions [25]. Many of these microfluidic systems consist of polydimethylsiloxane (PDMS), which are easy to fabricate and air permeable. However, the use of PDMSs can lead to problems when the low MIC of antibiotics has to be tested because of the absorption of small molecules and drugs during the testing [26,27,28].

In order to examine uncultured and therefore low concentrated samples in microfluidics, one challenge to be addressed is the on-chip filtration of the bacteria in small volumes to raise the concentration. This has to be done without clogging the system. This paper presents microfluidic capture to immobilize and detect the growth of *E. coli* which is suitable for chip-based ASTs. The basic principle behind this approach has already been used for the immobilization of human leukemic cells which were retained at a 5 µm gap and optically examined from above [29]. For the much smaller bacteria however, the design and manufacturing of the gap structure required a completely novel bypass cross-flow (BCF) approach.

## 2. Materials and Methods 

### 2.1. Microfluidic Chip Design

**Figure 1:**
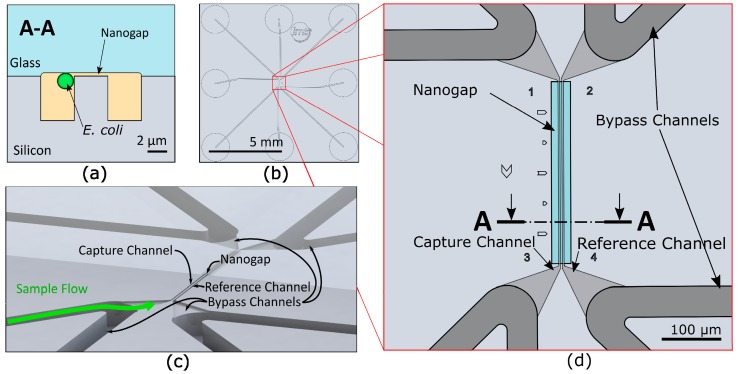
(**a**) Cross-section of the capturing area with a bacterium moving towards the nanogap, driven by a pressure difference between the channels. (**b**) Schematic top view of the 10 × 10 mm silicon-glass chip with eight microfluidic inlets and outlets at the bottom of the chip. (**c**) Schematic three-dimensional view of the capture features of the chip. (**d**) Detailed top view of the capture area in the red box with capture and reference channels, bypass channels, and nanogap area.

A schematic of the 10 × 10 mm BCF capture chip (Figure 1) shows the chip with inlet and outlet ports as well as the middle part of the chip, where the immobilization of bacteria is achieved at a nanogap between two 296 µm long parallel channels. They are connected by a shallow (300 nm) gap which is also 296 µm long and allows bacteria to be immobilized hydrodynamically by using different pressure levels on each side of the gap. The two channels are 3 µm apart and have a cross-section of 3 × 4 µm (see Figure 1a). Each end of these parallel channels is connected to a 50 × 50 µm bypass channel that is connected to one of the eight fluidic inlet and outlet ports on the bottom of the chip. This allows a quick loading of the gap area with bacteria and a fast exchange of the growth medium. By varying the pressure in the bypass channels, the flow through the capture channels and the gap region can be adjusted. This enables the capture as well as the release of bacteria.

### 2.2. Microfabrication

**Figure 2:**
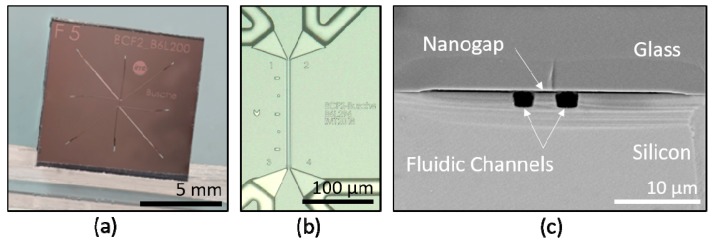
(**a**) Silicon-glass chips with a lateral dimension of 10 × 10 mm after wafer dicing. (**b**) Top view of the nanogap area and the bypass channels. (**c**) SEM image of a cross-section of the nanogap with reference and capture channels.

The chip used in this study was made of silicon and glass. The parallel fluidic channels were etched 4 µm deep into a silicon wafer using reactive ion etching in an inductive coupled plasma etcher (SPTS, Newport, UK) with an adapted mixed gas process (SF_6_/C_4_F_8_/Ar = 20/20/5) to get vertical sidewalls without any scallops [30]. The bypass channels were etched with a gas-switching process. A top view of the etched silicon structures is shown in Figure 2b. A glass wafer was locally etched with a depth of 400 nm with hydrofluoric acid to build the nanogap structure by anodic bonding with the silicon wafer. A cross-section of the bonded wafer is shown in Figure 2c. After bonding, fluidic connections were dry etched from the backside into the silicon. Then the wafers were diced to 10 × 10 mm chips, as shown in Figure 2a.

### 2.3. Experimental Setup

**Figure 3:**
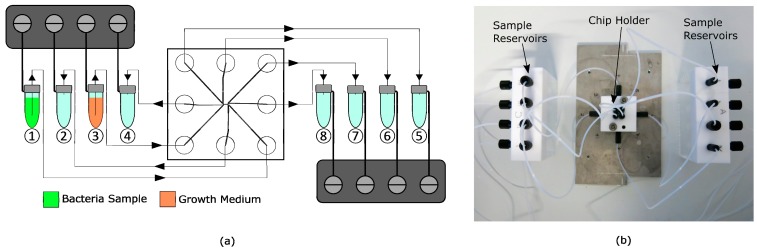
(**a**) Schematic of the fluidic setup with magnified bypass cross-flow (BCF) chip in the middle connected to sample and waste reservoirs and pressure controllers over the polytetrafluoroethylene (PTFE) tubing, driven by compressed air. Vials 1 and 3 are filled with bacteria sample and growth medium, respectively. Vials 2, 4, and 5–8 serve as waste reservoirs. (**b**) Fluidic setup with mounted chip in chip holder connected to the sample reservoirs.

The flow through the chip was achieved by eight pneumatic controllers (MFCS-EZ, Fluigent, Le Kremlin-Bicêtre, France), which were connected to eight 2 mL sample vials (Biozym Scientific GmbH, Hessisch Oldendorf, Germany), schematically shown in Figure 3a. The same tubing was used for connecting reservoirs with the microchip. A polytetrafluoroethylene (PTFE) filter membrane (Whatman, Maidstone, UK) between the tube outlets and the microchip ports was used to prevent clogging of the chip by particles bigger than 5 µm. Reservoirs and chip were placed on a fluorescence microscope (Axio Observer 3, Zeiss GmbH, Jena, Germany), surrounded by an incubator to maintain a cultivation temperature of 37 °C. Connecting components were designed and constructed out of PTFE. To avoid leakage, O-rings were placed between the fluidic chip and the PTFE holder, as shown in Figure 3b.

### 2.4. Bacterial Strain and Growth Medium

Cell capture and growth experiments were conducted using *E. coli* DH5-aplha cells containing the plasmid pPS858 [31]. This plasmid encodes the genes *bla* (beta-lactam-resistance), *aacC1* (gentamicin-resistance), and GFP (green fluorescent protein). Bacterial dilutions in the range of 10^5^ to 10^7^ cells/mL were prepared in Terrific Broth (TB) growth medium (Lionex GmbH, Braunschweig, Germany) containing 100 µg/mL carbenicillin (beta-lactam-antibiotic) and 10 µg/mL gentamicin-sulfate, which was also used as culture medium. Both antibiotics were added for selection of the used strain, carrying corresponding resistance genes. For growth inhibition experiments, a 200 µg/mL amount of kanamycin was added to the medium, which is 100-fold above the minimum inhibition concentration (MIC) of kanamycin (2 µg/mL) [32]. Prior to experiments, the bacteria suspension and TB medium were filtered with a 5 µm syringe filter (Whatman, Maidstone, UK). Due to the storage at 4 °C, the bacteria suspension and medium were additionally pre-incubated 1 h at 37 °C before their use in the growth experiments. No further treatment was applied to the bacteria.

### 2.5. Calculation of Growth Rates

Specific growth rates *µ* and associated standard deviation were calculated using linear fits to logarithmic cell length over time (*t*) plots. Additionally, doubling times *t_d_* with corresponding standard deviation (Gaussian error propagation) were determined as follows:

(1) $$\mu =\frac{\Delta \ln\left(\mathrm{Length}\right)}{\Delta t}$$

(2) $$t_{d}=\frac{\ln\left(2\right)}{\mu }$$

## 3. Results and Discussion

### 3.1. Fluidic Simulations of Shear Forces

For a quick loading procedure, a high flow velocity through the channels is desired. Researchers must ensure that the shear stress acting on the bacteria surface during capturing is below 1250 Pa, otherwise the bacteria is damaged and their proliferation is stopped [33]. Therefore, Computational fluid dynamics (CFD) simulations were carried out using ANSYS Fluent (Version 17.0, ANYSY Inc., Canonsburg, PA, USA).

**Figure 4:**
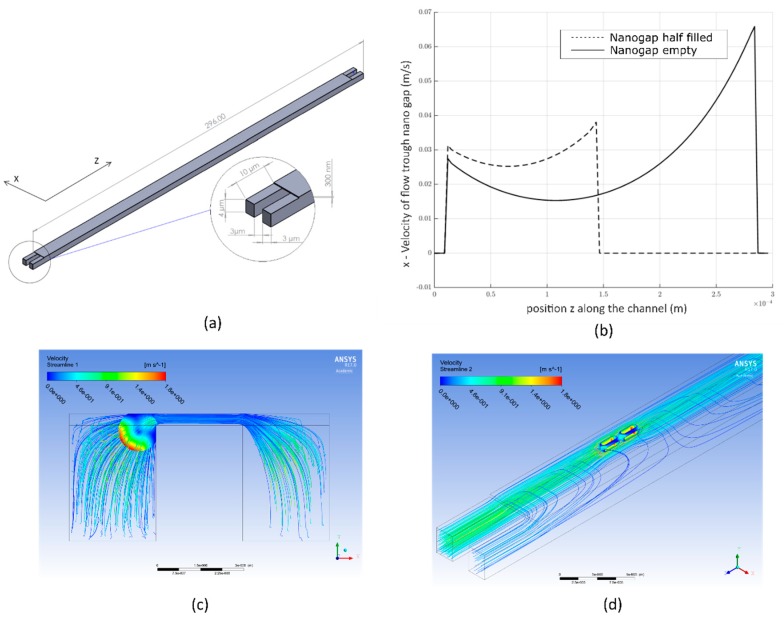
(**a**) Geometry used for the simulation of flow-induced shear stress on the *E. coli* cell with coordinate system. (**b**) Velocity of the flow through the nanogap in the x-direction over the channel length. (**c**) Cross-sectional view into the channels at the gap area with streamlines around the fixed *E. coli* model. (**d**) Isometric view into the nanogap area with streamlines flowing around the bacteria.

An *E. coli* bacterium model (0.7 µm diameter and 3.3 µm length) was placed at the nanogap area, as shown in Figure 4c, and the flow was adjusted so that the resulting force on the bacteria was directed to the gap. Two local maxima of the shear stress were found on the surface of the bacteria at the nanogap, as shown in Figure 4c,d. For the maximum desired pressure difference of 2 bar for the channel inlet to outlet, the shear stress stayed below 1250 Pa at a maximum average shear stress over the bacterial surface of 485 Pa. A flow rate of 0.2618 µL/min was obtained. With a concentration of 10^4^ *E. coli* cells/mL, it would theoretically take 10 min to fill the system with 100 bacteria if every *E. coli* was captured at the gap area.

To obtain the distribution of the velocity of the flow through the nanogap along the channel, another flow simulation was carried out. The pressure-driven flow through the gap reached local maxima at the channel ends (z = 0 and z = L), where the pressure difference between the channels was the highest, as shown in Figure 4b when the channel is empty. This flow profile prefers capturing the bacteria at the end (z = L) of the channel and thereby continuously blocks the gap, leading to continuously reducing the active gap length from the end to the beginning (z = 0). Therefore, the flow profile continuously changes as the gap is increasingly blocked with bacteria from the end to the front, as shown in Figure 4b. To prevent clogging, the channels were designed in dimensions to enable larger particles to pass through the channels even when bacteria were captured, as shown with the streamlines in Figure 4d.

### 3.2. Cell Capture Experiments

**Figure 5:**
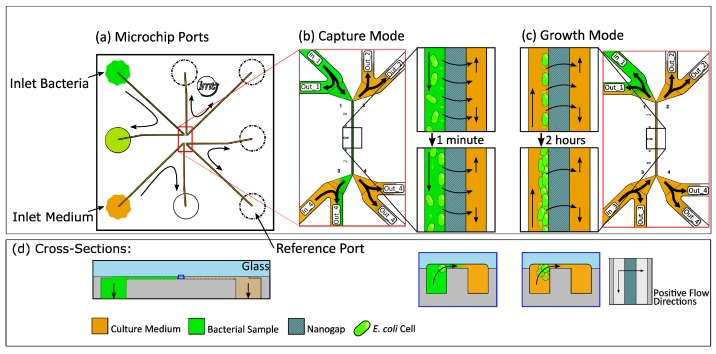
(**a)** Schematic top view and (**d**) cross-section with flow conditions for (**b**) capture and (**c**) growth in the BCF chip with black arrows indicating the fluidic flow directions.

Before growth experiments could be executed, the right pressure settings of the connected pressure controllers had to be evaluated. The conducted experiments consisted of capture and growth phases. For the capture phase, the pressure at the pressure controllers was regulated so that the bacteria sample flowed through the bypass channel 1 from Inlet 1 to Outlet 1. This resulted in a higher pressure at the entrance of the detection channel in the system than in its outlet. The bacteria flowed into the detection channel and were captured at the nanogap by a pressure difference towards the reference channel, schematically shown in Figure 5. Capturing a sufficient number of cells at the nanogap took 3–4 min during typical growth experiments, independent of concentrations varying between 10^6^ cells/mL and 10^7^ cells/mL. The experimentally obtained pressure settings for the capture are shown in Table 1.

Real-time microscopy allowed ideal timing to switch the pressure settings to growth mode. Including the subsequent adjustment of the imaging setup, the preparation time between pre-incubation and the beginning of detection took 15 min on average. The pressure difference between the detection bypass channels was inverted, causing a flow direction from growth medium to bacteria bypass through the detection microchannel. In some cases, pressures were adjusted in more than one step to avoid losing too many cells by flushing. For the duration of 4 h, growth mode was kept constant and the microchip was imaged every 30 s to monitor whether the cells were sufficiently retained and were growing at the nanogap. The pressure values determined to be suitable for the capture and a 4 h retention of the bacteria are summarized in Table 1.

**Figure 6:**
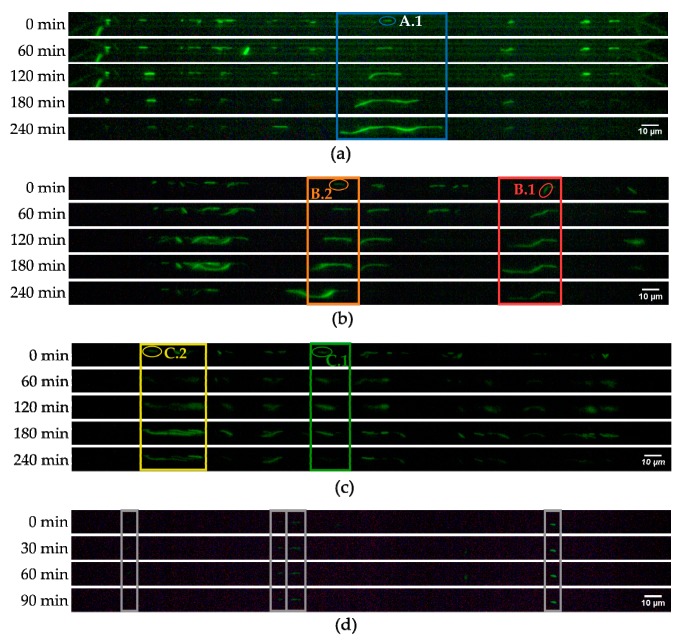
Fluorescent microscope images (20× magnification) showing *E. coli* immobilized at the nanogap at 37 °C with Terrific Broth (TB) medium flowing through the detection channel over 4 hours. (**a**–**c**) Three exemplary growth experiments designated A, B, and C. Measured cells are numbered within each experiment. (**d**) Negative control with addition of antibiotics (200 µg/mL kanamycin). Marked are measured cells.

The evaluation of the fluorescence pictures was done with ImageJ (Version 1.8.0, NIH, Bethesda, MD, USA). (A scale was determined with known dimensions of the microchip. Length measurements of cell agglomerates were performed after adjusting the fluorescence contrast.) The results are shown in Figure 6. The different cell agglomerates were measured. The density of trapped cells turned out to be a limiting factor in the manual graphic analysis. High cell densities in the channel caused cell overlapping, further enhanced by cell growth. This also resulted in less stable immobilization and therefore loss of cells during growth experiments. Thus, the number of captured cells was reduced to avoid overlapping of the bacteria.

### 3.3. Growth Experiments 

After pre-incubation, the bacterial dilution containing 10^6^ cells/mL and culture medium were connected to the microfluidic setup. All growth experiments were carried out at 37 °C with TB growth medium. After initially applying high pressure of 400 mbar for 2 min to fill the tubing and channels, the system was set to capture mode until the bacteria in the detection channel still had sufficient space between each other. After filling the channel with bacteria, the pressure setup was switched to growth mode and no new bacteria were loaded into the channel. An overall lower pressure at both bacteria bypass ports generated a flow direction from medium to bacteria bypass through the detection microchannel. This ensured a fresh medium supply to the growing cells. Additionally, the application of only low pressures to the reference ports led to a pressure difference (Δp = 150 mbar) between detection and reference microchannel, resulting in suction through the nanogap and thereby immobilization of cells. Imaging settings (exposure time, gain, and LED intensity) were set individually to allow optimal fluorescence detection. Every 30 s, a fluorescent image was taken with a custom-made time-lapse setup.

Due to the marginal flow rate of fresh medium, cells were not separated after division. This allowed the observation of single cells developing into long cell chains. The increase of total chain length was analyzed over time. Within single experiments, a high diversity in chain-growth behavior could be observed. A typical heterogeneity was observed in all conducted experiments. The already small number of trapped cells inside the microchip was considerably reduced by non-proliferating and dying cells. With cell death, fluorescence faded during experiments. Moreover, part of the living cells did not proliferate until the last hour of the experiment. Only a few cells proliferated and led to growing chains directly from the beginning. This can be explained by phenotypic heterogeneity, enhanced by stressful conditions and the statistical effect of extremely small populations [34].

Nevertheless, it was possible to analyze cell chain growth, with reproducible results within and between experiments. Monitoring the length of selected chains over time indicated a phase of exponential growth starting around 15 min after the beginning of the experiment and stagnation after about 2 h, typically terminating in length reduction and fading fluorescence.

We further present five exemplary cells, which were investigated thoroughly while developing into a (growing) chain. Plotting chain lengths over time shows significant growth starting 15 min after loading for all of them. However, the growth of chain length diverges immensely after 90 min. Thus, only the period between 15 min and 90 min was taken into account for further analysis. 

**Figure 7:**
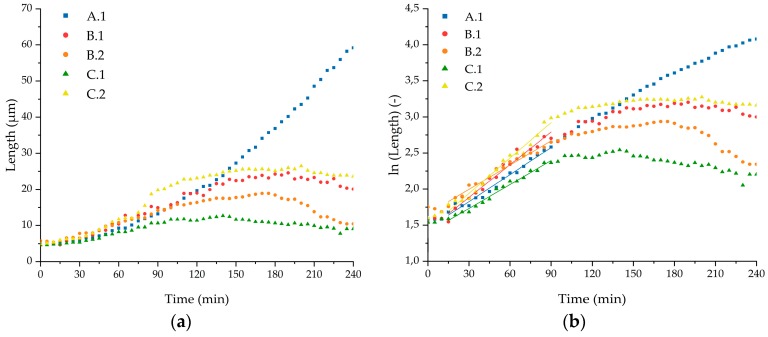
(**a**) Length development over time of selected *E. coli* cells. (**b**) Logarithmic length of *E. coli* cells over time with linear fits for specific growth rate determination between 15 min and 90 min of growth experiments in TB medium at 37 °C.

The specific growth rate was calculated by logarithm of length over time plots. Here, within the window of 15 min to 90 min, the graphs show a linear trend (Figure 7b). Linear fits lead to specific growth rates between 0.011 min^−1^ and 0.016 min^−1^ with an average specific growth rate of 0.013 min^−1^. Additionally, doubling times were calculated (= ln(2)/specific growth rate), resulting in a mean value of 53 min. The results of the individual cells and mean values are summarized in Table 2. Standard deviations were determined by linear fits (specific growth rate) and Gaussian error propagation (doubling time).

### 3.4. Antibiotic Treatment Experiments

**Figure 8:**
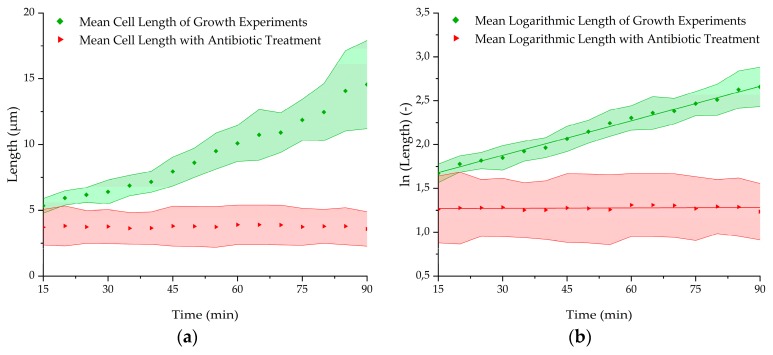
(**a**) Mean length growth, (**b**) mean logarithmic length growth with and without 200 µg/mL kanamycin in TB medium at 37 °C at the nanogap. Statistical significance was tested using two-sample t-test with a significance level of 0.01. Calculated p-values (<0.01) indicate that the growth rate of the antibiotic treated bacteria (n = 4) differs significantly from the population without treatment (n = 5).

After testing the growth of *E. coli* in the microchip in a capsuled environment, the influence of an antibiotic with an inhibitory effect on the bacteria was used to evaluate if the proliferation could be hindered in a short time. Therefore, a growth experiment with 200 µg/mL kanamycin added to the TB medium was carried out. The capture was done with 10^6^ cells/mL and at 37° C as previously performed. After 3 min of cell capturing, the pressure parameters were switched to growth mode. Every 30 s, a fluorescent image was taken over a period of 90 min. After the experiments, the fluorescent images were analyzed and the bacterial chain length was measured. Figure 8 shows the results of these experiments in direct comparison to the average length of the experiments in the antibiotic-free medium above. It is recognizable that the kanamycin is hindering the *E. coli* to proliferate as the length is not enlarging over time, whereas the bacteria in the kanamycin free-medium start growing directly from the beginning with a mean growth rate of 0.0131 min^−1^ and a doubling time of 53 min, respectively (Table 2). The results show that a difference in growth behavior can be seen already 15 min after finishing the loading procedure. This makes this BCF principle capable of holding bacteria back in a desired area while supplying antibiotics or other drugs to the captured bacteria in short time.

## 4. Conclusions

In this study, we demonstrated a novel nanogap capturing principle, which enables the immobilization of vivid *E. coli* cells for their later use in lab-on-a-chip optical-detection devices. In simulations and experiments we showed that this capture method does not damage the bacteria and allows proliferation. By using bypass channels, antibiotics or other drugs can be transported into the capture channels within seconds. Thus, the influence on the bacterial proliferation of these drugs can be investigated. Instead of image analysis requiring fluorescence microscopy (and fluorescent microorganisms), this capturing principle could be achieved within many channels in parallel to measure refractive index change due to bacterial growth by the diffraction of a laser beam that irradiates the grating. This diffractive approach has so far only been used for the label-free detection of proteins [35] and will be investigated in our future research for fast antibiotic susceptibility testing (AST). In addition, such a principle can be applied in phase-contrast investigations in which bacteria are to be immobilized without markers. It could thus prove also as a helpful tool in persistence research where single-cell growth behavior is observed.

## Figures and Tables

**Table 1:** Applied pressure settings to inlets (*p_In1_–p_In4_*) and outlets (*p_Out1_–p_Out4_*) of the microchip for growth experiments in capture and growth modes.

Mode	Pressure Applied (mbar) at the Inlets and Outlets							
	*p_In1_*	*p_Out1_*	*p_In2_*	*p_Out2_*	*p_In3_*	*p_Out3_*	*p_In4_*	*p_Out4_*
Capture	200	195	195	170	40	40	50	50
Growth	190	180	200	185	40	40	40	40

**Table 2:** Specific growth rate of each immobilized and observed *E. coli* cell at the nanogap at 37 °C with TB medium flowing around the bacteria.

Cell	Specific Growth Rate (min^−1^)	Deviation (min^−1^)	R^2^ (Fit)	Doubling Time (min)	Deviation (min)
A.1	0.0125	0.0005	0.9790	56	2
B.1	0.0151	0.0007	0.9694	46	2
B.2	0.0112	0.0004	0.9789	62	2
C.1	0.0111	0.0004	0.9859	63	2
C.2	0.0164	0.0007	0.9773	42	2
Mean	0.0131	0.0003	0.9928	53	1
